# Sex differences in the chronic autoimmune response to myocardial infarction

**DOI:** 10.1042/CS20243091

**Published:** 2025-06-17

**Authors:** Erin B. Taylor, Luciano D. Mendoza, Jayla D. Sandifer, Jemylle G. Morato, Nikaela M. Aitken, Katherine R. O'Quinn, Indu Raman, Chengsong Zhu, Robert W. Spitz, John E. Hall, Alan J. Mouton

**Affiliations:** 1 Department of Physiology and Biophysics, University of Mississippi Medical Center, Jackson, MS 39216, U.S.A; 2 University of Texas Southwestern Medical Center, Dallas, TX 75390U.S.A

**Keywords:** autoimmune disease, inflammation, heart failure, myocardial infarction, sex differences

## Abstract

Myocardial infarction (MI) causes a robust inflammatory response, which is necessary for remodeling and scar formation of the infarcted left ventricle (LV). However, this can lead to chronic systemic inflammation and persistent autoimmune responses. In this study, we analyzed sex differences in the inflammatory autoimmune response to chronic MI. MI was induced by permanent left coronary artery ligation in adult male and female C57BL/6J mice for one, four, and eight weeks. Both sexes exhibited similar declines in LV function. Females had higher levels of total immune cells and T cells in the infarct and remote area at D7 post-MI, and B cells at D56. MI increased levels of pro-inflammatory cytokines (*Il1b, Il6, Tnf, Ccl2, Ifng, Il18*) in the LV infarct that peaked at one week, which was exaggerated in females for *Il6, Ifng, and Il10*. In the remote LV, females had higher levels of *Il6, Tnf, Ccl2,* and *Il18*. MI increased spleen mass in females only, and splenic cytokines were higher in females at several time points, including *Il1b, Il12a, Il10, Ifng, Il18, Ccl2,* and *Il4*. IgG and IgM deposition in the LV infarct increased over time in both sexes, but more so in females. In the remote area, both sexes had increased IgG and IgM at eight weeks. Plasma IgM was higher in females at one, four, and eight weeks post-MI compared with males. Plasma IgG and IgM autoantibodies were detected in males and females after MI, but the number of autoantibodies displaying reactivity to autoantigens was much higher in females, particularly at week 8. In summary, MI leads to the development of systemic and myocardial autoimmune activation, which is more pronounced in females.

## Introduction

Myocardial infarction (MI) is one of the leading causes of heart failure and mortality due to cardiovascular disease worldwide [1]. MI leads to significant remodeling of the left ventricle (LV), which involves a robust local and systemic inflammatory response [2,3]. The early inflammatory phase is characterized by infiltration of innate immune cells, including neutrophils and monocytes that differentiate into tissue macrophages. Adaptive immune cells, including B and T lymphocytes, also play a role in the early response, although their specific functions are less well-defined [4–10]. Furthermore, chronic LV remodeling due to MI is associated with persistent systemic inflammation [11,12], which has been linked to chronic activation of autoreactive T and B cells and increased production of autoantibodies [13,14].

Recent evidence has established a link between autoimmune disease and cardiovascular disease, particularly in females. The prevalence of autoimmune disease, in which there is a breakdown of central and peripheral tolerance mechanisms, leading to immune cell hyperreactivity and end-organ damage, has risen in recent decades to its current estimate of ~10% of the population, disproportionately affecting women [15]. Autoimmune disease has been linked to increased risk of MI and adverse post-MI outcomes in female patients, including recurrent MI, development of heart failure, and mortality [16]. Furthermore, MI can promote the development of autoimmune characteristics in patients without pre-existing autoimmune disease [17,18].

The purpose of this study was to investigate local and systemic inflammatory and autoimmune responses after chronic MI and potential sex differences. We found evidence of adaptive and autoimmune responses in adult male and female mice, particularly at more chronic time points, which was more pronounced in females. Our results highlight the importance of sex-specific pathways of cardiac remodeling and systemic inflammation following MI and suggest that females may be more susceptible to developing autoimmune characteristics following MI.

## Methods

### Animals

C57BL/6 J mice (*n* = 87) were purchased from Jackson Laboratories (Bar Harbor, ME). Mice were acclimated to the vivarium for at least one week prior to surgery. Mice were maintained on a 12 h light/dark cycle in temperature-controlled rooms (73–76°F) with access to chow and water *ad libitum*. All studies were performed at the University of Mississippi Medical Center with approval from the Institutional Animal Care and Use Committee (protocol #1371) and in accordance with National Institutes of Health Guide for the Care and Use of Laboratory Animals.

### Surgical induction of MI

MI was induced by permanent ligation of the left coronary artery in adult (16–20 week old) male and female C57BL/6 J mice as previously described [19–21]. Mice were anesthetized (2% isoflurane), intubated using a rodent ventilator (Harvard Apparatus), and the intercostal muscles exposed. The heart was exposed between the third and fourth rib, and the left coronary artery was ligated with an 8–0 suture. Blanching of the heart below the suture was used to confirm successful MI. The surgical site was then sutured with 5–0 suture, and the mouse was extubated and placed on a warming pad for recovery. At each experimental end-point, mice were anesthetized with 2% isoflurane, blood was taken from the carotid artery, and the heart was quickly removed. For measurement of infarct size, mid-LV histological sections were stained with picrosirius red and analyzed with ImageJ. Representative H&E images of the LV are displayed in Supplementary Figure S1.

### Echocardiography

Cardiac function and structure were assessed using ultrasound echocardiography (VEVO 3100, VisualSonics). Mice were anesthetized (2% isoflurane), and the ultrasound was performed in the supine position. Long-axis and short-axis B and M-mode images were acquired. The images were then analyzed using VEVOLab imaging software. The following parameters were measured: wall thickness (LVAWd and LVPWd), LV diameter at systole and diastole (LVIDs and LVIDd), end-systolic and end-diastolic volume (ESV and EDV), and ejection fraction (EDV-ESV/EDV × 100).

### Preparation of cells for flow cytometry

Blood was collected from the retroorbital plexus at the conclusion of the study. The blood was centrifuged at 350×g for 5 min to isolate plasma. Erythrocytes were lysed by adding 10× volume of 1X PharmLyse (BD Biosciences, San Jose, CA). After incubation for 5 min at room temperature, the blood was centrifuged at 200×g for 5 min. The pelleted peripheral blood leukocytes (PBL) were washed with 1X PBS, 2% FCS, and centrifuged at 350×g for 5 min. Spleens were homogenized using the Spleen Dissociation Kit (Miltenyi Biotec, Bergisch Gladbach, Germany) and the GentleMACS Octo Dissociator (Miltenyi Biotec) according to the manufacturer’s instructions. Remote and infarcted heart tissue samples were diced into small pieces in 5 ml of RPMI media containing 200 U/ml DNase and 10 mg/ml collagenase IV. The diced pieces and RPMI were transferred to a GentleMACS tube and further homogenized using the mouse neonatal heart dissociation protocol on the GentleMACS Octo Dissociator. After dissociation, the resulting homogenates were filtered through a 70 μM cell strainer and washed with 1X PBS containing 2% FCS and 2 mM EDTA, and the single-cell suspension was centrifuged at 300×g for 10 min. Erythrocytes were lysed in 3 ml of 1X PharmLyse for 5 min at room temperature and washed with 27 ml of 1X PBS containing 2% FCS and 2 mM EDTA. After centrifugation at 350×g for 5 min, the cells were used for flow cytometric analyses.

### Flow cytometry

Flow cytometric analyses were performed as previously described by our laboratory [22,23]. Briefly, cells were first washed and resuspended in 1X PBS, 2% FCS, and 0.09% sodium azide at a concentration of 2 × 10^7^ cells/ml. 1 × 10^6^ cells (50 μl) were aliquoted into a flow cytometry tube and incubated with 0.25 μg of anti-mouse CD32/CD16 (FcR block, BD Biosciences) for 5 min on ice. Cells were stained with either isotype control antibodies or antibodies shown in Supplementary Table S1 for 30 min on ice protected from light. All antibodies were diluted 1:200 in 1X PBS, 2% FCS, and 0.09% sodium azide. Cells were analyzed using a BD FACSymphony A3 analyzer, and a total of 100,000 events were captured per sample. Data were analyzed using FlowJo version 10.10.0 (BD Biosciences).

### Hemavet analysis

Blood was collected at euthanasia into tubes containing EDTA. Blood samples were analyzed using the Hemavet 950FS Auto Blood Analyzer (Drew Scientific) to determine circulating concentrations of leukocytes, lymphocytes, neutrophils, and monocytes.

### Real-time polymerase chain reaction

Gene expression in LV infarct, remote, and spleen tissue was assessed by real-time PCR. RNA was extracted from snap frozen tissue samples. For cDNA synthesis, 1 µg RNA was used for reverse transcription (iScript Reverse Transcription Super-Mix; Bio-Rad). The PCR reaction was then performed with 50 ng cDNA using SYBR Green Master Mix (Bio-Rad) and primers specific (Integrated DNA Technologies) for the following genes: *Il1b*, *Il6*, *Tnf*, *Ccl2*, *Ifng*, *Il18*, *Il12a*, *Il10*, and *Il4. Actb* was used as a housekeeping gene. Fold change values were calculated using the 2^-ΔΔCt^ method.

### Immunofluorescence

LV IgG and IgM deposition were quantified using immunofluorescence performed with the Opal 6-Plex Detection Kit (Akoya Biosciences). Mid-LV sections were fixed in zinc formalin overnight, paraffin-embedded, and sectioned at 5 µm on microscope slides. Slides were then rehydrated, blocked for 10 min, and incubated overnight at 4°C with either goat anti-mouse IgG-HRP (1:500; Thermofisher #31430) or goat anti-mouse IgM-HRP (1:100; Thermofisher #PA1-84383). The next day, slides were incubated with the Opal 520 fluorophore, counterstained with DAPI, and mounted. IgG or IgM fluorescence was imaged at 20X with the Mantra^TM^ system (Perkin Elmer) on a fluorescent microscope in the FITC channel with the investigator blinded to the samples. Fluorescence intensity was then quantified in the infarct and remote regions using inForm^TM^ software (Perkin Elmer).

### ELISAs

Total IgM and IgG levels (Alpha Diagnostic International; San Antonio, TX), troponin, IL-10, C-reactive protein, and interferon-gamma (R&D Systems; Minneapolis, MN) were detected in plasma using commercially available ELISA kits according to the manufacturer’s instructions.

### Plasma autoantigen array

Autoantigen microarrays were manufactured in the Microarray & Immune Phenotyping core Facility of University of Texas Southwestern Medical Center, Dallas, TX, U.S.A. A selection of 120 autoantigens was made based on published literature, prior known autoantibodies in various immune-related diseases, cancer, allergic disease, and so on. Eight positive control proteins (Ig control 1:2, Ig control 1:4, Ig control 1:8, Ig control 1:16, anti-Ig control 1:2, anti-Ig control 1:4, anti-Ig control 1:8, anti-Ig control 1:16) were also imprinted on the arrays as positive controls. There are 16 identical arrays on each slide. Each array contains 124 autoantigens and 4 internal controls. For sample processing, each slide was processed with 15 samples and PBS as a negative control. Mouse samples were first treated with DNase I to remove free DNA and then applied onto autoantigen arrays with 1:50 dilution. The sample was incubated with the autoantigen array, and the autoantibodies binding to the antigens on the array were detected with laser wavelengths 532 nm (cy3 labeled anti-IgG) and 635 nm (cy5 labeled anti-IgM) and generated TIFF images. Genepix Pro 7.0 software was used to analyze the images and generate the Genepix report (GPR) files (Molecular Devices, Sunnyvale, CA, U.S.A.). The net fluorescent intensity (NFI) of each antigen was generated by subtracting the local background and negative control (phosphate-buffered saline or simplified as PBS) signal. The signal-to-noise ratio (SNR = (foreground median − background median)/standard deviation (background)) was also generated for each antigen to resolve true signal from background noise. NFI was normalized by robust linear model using positive controls with different dilutions. To avoid outliers in either NFI or SNR, autoantibody score (Ab-score) was also calculated by log 2 ((NFI × SNR) + 1).

### Statistics

Data are represented as mean ± SEM. For comparison of sex differences across time, two-way ANOVA with Tukey’s post-hoc analysis was used.

## Results

### Survival rate, morphometric data, and cardiac function in post-MI male and female

**Figure 1:**
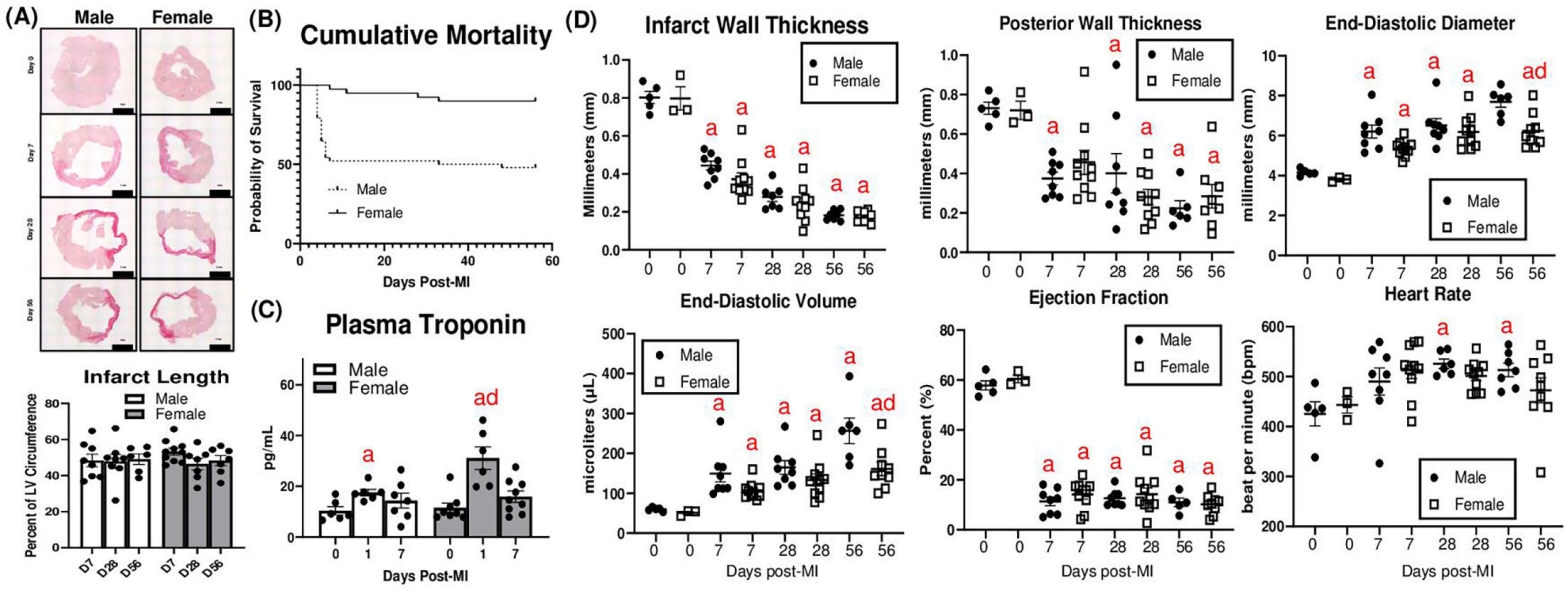
Infarct size, mortality, and post-MI cardiac function. (**A**) Representative LV midsections stained with picrosirius red and infarct length expressed as percentage of total LV circumference. (**B**) Cumulative mortality (D7, D28, D56 groups combined). (**C**) Plasma troponin measurements at D1 and D7 after MI. (**D**) Echocardiography parameters. a*: P*<0.05 vs. day D0, b*: P*<0.05 vs. D7, c*: P*<0.05 vs. D28, d*: P*<0.05 male vs. female. *N* = 6–10 mice per group.

Male and female adult mice were studied at days (D) D0 (no MI), D7, D28, and D56. No differences in infarct length were observed by picrosirius red staining (Figure 1A). Cumulative mortality was significantly higher in males than females (Figure 1B). Plasma troponin is typically elevated transiently after MI and often reflects the degree of cardiac injury [24]. At D1 post-MI, males and females exhibited increases in plasma troponin, which was higher in females; however, troponin returned to basal levels in both sexes by D7 (Figure 1C). By echocardiography, males and females exhibited similar decreases in thickness of the LV anterior (i.e., infarcted) and posterior walls (Figure 1D). However, males exhibited more LV dilation as assessed by the end-diastolic diameter and end-diastolic volume, particularly at D56. Males exhibited increases in heart rate at D28 and D56, which was not observed in females. Both sexes exhibited similar decreases in ejection fraction. LV mass was elevated at all time points in both sexes, particularly at D56 (Supplementary Figure S2**)**. Increased lung mass, an indicator of pulmonary edema [19–21], was observed at D7 post-MI in both sexes. However, lung mass was not significantly different at D28 and D56 compared with D0 in males but remained elevated in females at D28 and D56 (Supplementary Figure S2).

### Changes in immune cell populations after MI

**Figure 2:**
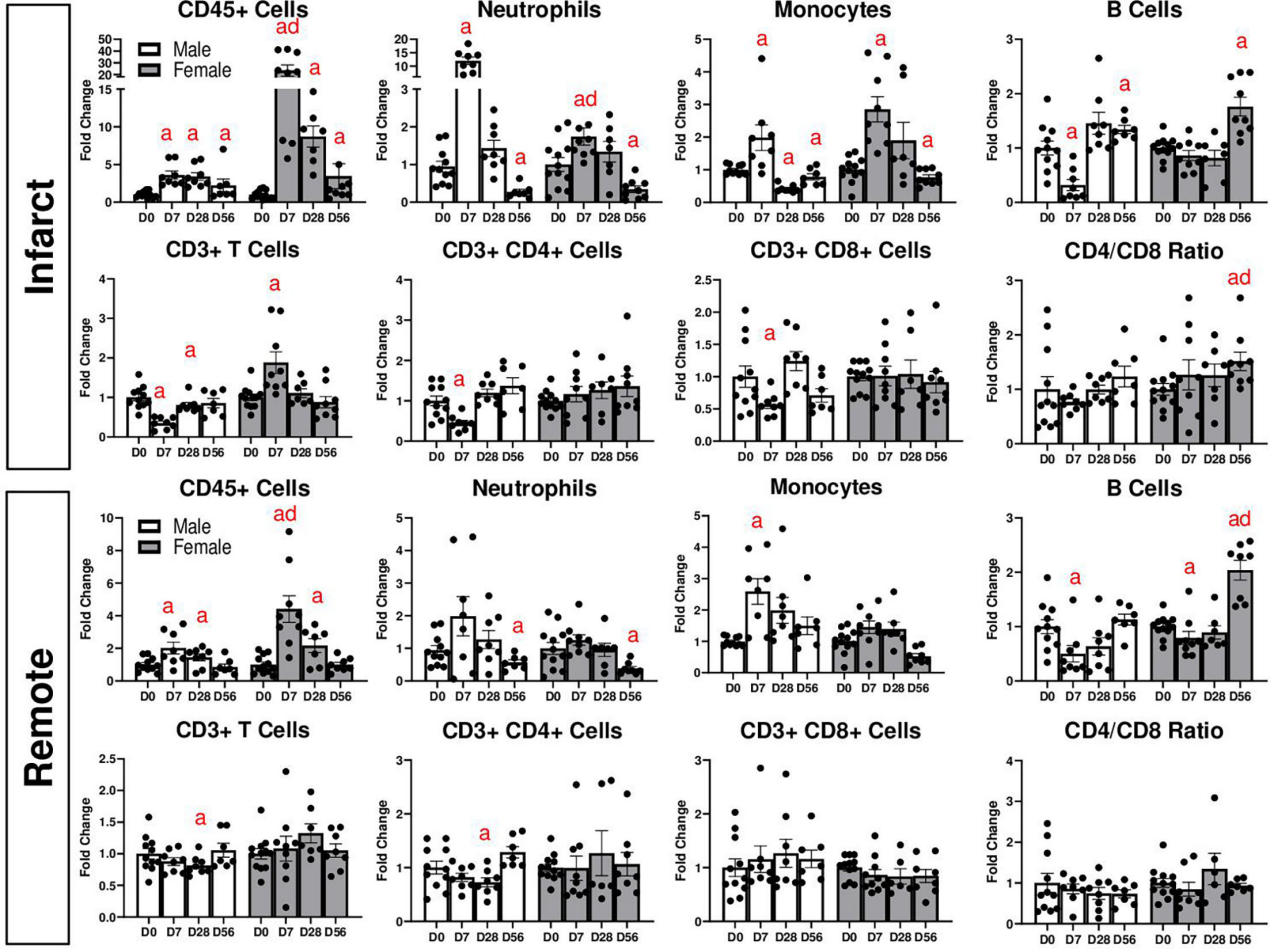
Flow cytometry analysis of post-MI LV tissue for (**A**) infarct area and (**B**) remote area. a: *P*<0.05 vs. day D0, d*: P*<0.05 male vs. female. *N* = 6–10 mice per group.

We measured immune cell populations from the heart, spleen, and peripheral blood at each time point by flow cytometry (representative gating in Supplementary Figure S3 and S4). In the infarct area, CD45^+^ leukocytes were robustly increased in females compared with D0, and to a lesser extent in males, at D7 and D28, and began to decline by D56 in both sexes (Figure 2). Neutrophils were increased in the infarct in both males and females at D7, to a significantly higher extent in males. Infarct neutrophils returned to baseline levels by D28 and were decreased at D56 in both sexes compared with D0. Infarct monocytes were increased to a similar extent at D7 in both males and females but were significantly lower in males at D28 (compared with D0 and to D28 females). At D56, infarct monocytes were decreased in both sexes compared with D0. CD3^+^ T cells followed different patterns in males and females. Females exhibited increased CD3^+^ T cells in the infarct at D7, but no changes were observed between CD4^+^ and CD8^+^ populations. In contrast, males had decreased levels of CD3^+^ T cells in the infarct at D7, which was true for both CD4^+^ and CD8^+^ subpopulations. At D28, CD3^+^ cells remained decreased in males and returned to basal levels in females. Both sexes exhibited no differences in infarct CD3^+^ T cell percentages at D56, although the CD4/CD8 ratio was increased in females. In males, infarct B cells were decreased at D7, returned to basal (D0) levels by D28, and increased at D56. In females, infarct B cells were not changed at D7 or 28 but increased at D56 to a higher degree than males.

We also observed changes in immune cells in the remote (non-infarcted) area of the heart (Figure 2B). Similar to the infarcted area, remote CD45^+^ immune cell levels were increased in both sexes, to a higher degree in females. Neutrophils were not significantly different from D0 at D7 or 28 in either males or females but were significantly decreased compared with D0 animals at D56. Monocytes were increased in females, but not males, at D7. Remote CD3^+^ cells were not altered by MI, but there were higher numbers of CD3^+^ cells in females at D28 compared with males. There were no changes in the CD4/CD8 ratio for either sex in the remote area. B cells were not changed at D7 or 28 in females but were increased at D56 compared with D0 and compared with males at D56. No changes in B cells were observed in males.

**Figure 3:**
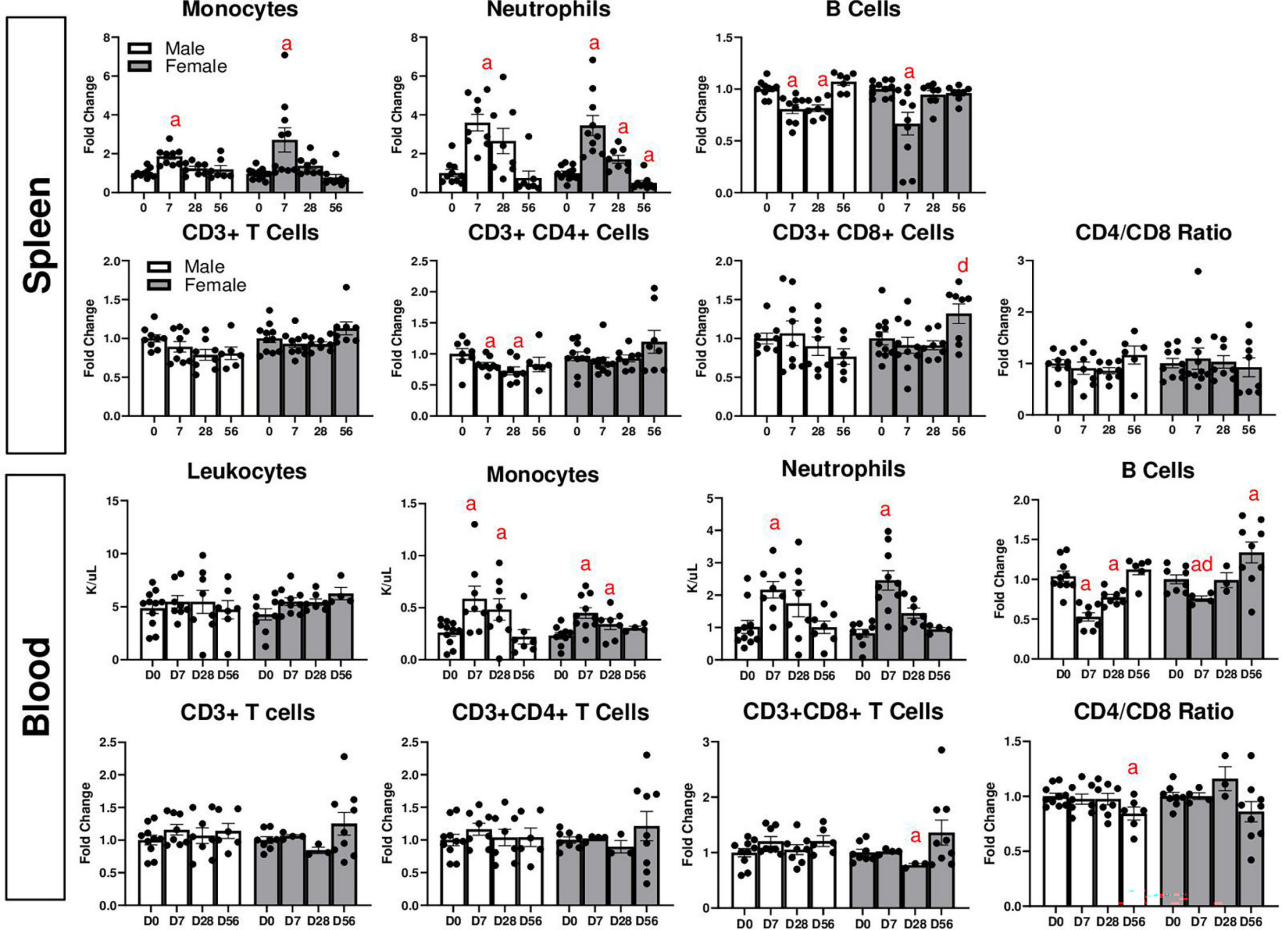
Flow cytometry analysis of spleen tissue after MI. a: *P*<0.05 vs. day D0, d: *P*<0.05 male vs. female. *N* = 6–10 mice per group.

MI has also been associated with changes in splenic and circulating immune cells [25,26], which we found to be true in our study (Figure 3). B cells were decreased in the spleen in both sexes at D7 and in males at D28. Monocytes were increased at D7 in both males and females, then returned to basal levels. Neutrophils were increased at D7 in both sexes and remained elevated in females at D28 but were decreased at D56 in females. CD3^+^ cells were not significantly altered by MI but were higher in female spleens at D56 compared with males at the same time point. CD4^+^ T cells were decreased in male spleens at D7 and D28 compared with D0, and female spleens had higher levels of CD4^+^ cells at D28 compared with males. Splenic CD8^+^ cells were not affected by MI but were higher in female spleens at D56 as compared with males. No changes in the CD4/CD8 ratio were observed in the spleen for either sex.

We also observed changes in circulating immune cell populations (Figure 2). While no changes were observed in total circulating leukocytes, monocytes were increased in both sexes at D7 and D28, and neutrophils were increased at D7. As MI is known to cause acute lymphopenia [27], we detected decreases in circulating B cells at D7, which was more severe in males and remained decreased at D28. In contrast, B cells returned to basal levels in females by D28 and were elevated at D56. There were no changes in total CD3^+^ T cells or CD4^+^ T cells. However, there was a slight reduction in CD8^+^ T cells in females at D28 and a decrease in the CD4/CD8 ratio in males.

### Changes in LV cytokines after MI

**Figure 4:**
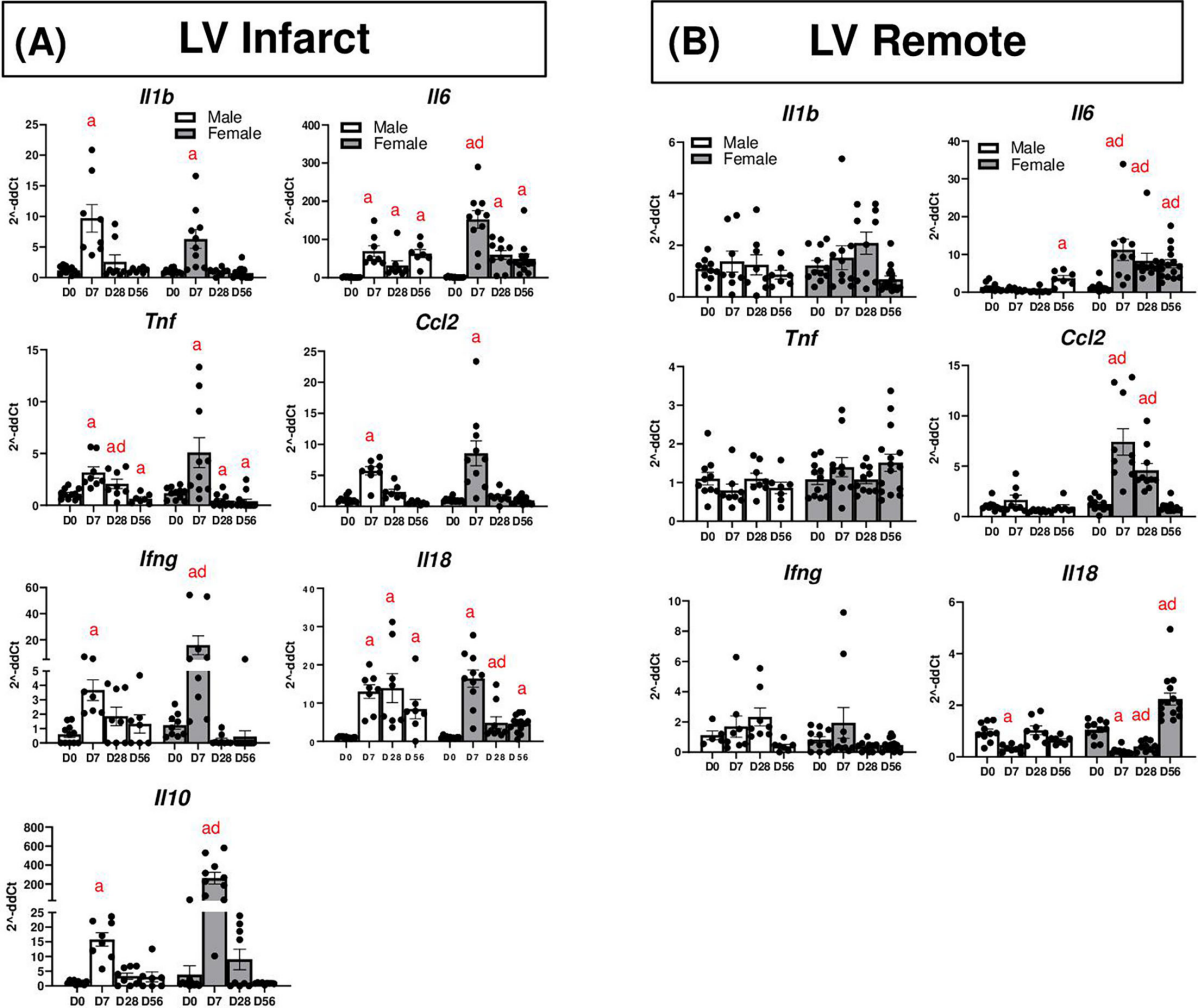
Gene expression analysis of inflammatory cytokines in the LV infarct (**A**) and remote (**B**) after MI. a: *P*<0.05 vs. day D0, d: *P*<0.05 male vs. female. *N* = 6–10 mice per group.

We then sought to assess changes in LV cytokines after MI, both in the infarct and remote areas. We analyzed gene expression of several major pro-inflammatory cytokines, including *Il1b*, *Il6*, *Tnf*, *Ccl2*, *Ifng*, and *Il18*, as well as *Il10*, which is predominately an anti-inflammatory cytokine [28]. In both sexes, all were significantly elevated at D7 post-MI in the infarct area (Figure 4A). However, this increase was higher in females for *Il6*, *Il10*, and *Ifng*. Cytokine expression was also analyzed at D1 post-MI (Supplementary Figure S5), and *Ccl2* was higher in males at D1. In both sexes, *Il1b*, *Il10*, *Ccl2*, and *Ifng* returned to basal levels by D56. However, *Il6* remained at D28 and D56, although to a lesser extent than at D7, in both sexes. *Tnf* remained elevated in males at D28, while decreasing in females. However, *Tnf* was not different between sexes at D56. *Il18* remained elevated at D28 in males but was decreased compared with D7 in females. At D56, *Il18* remained elevated compared with D0 in both sexes.

MI was also associated with changes in cytokines in the remote area (Figure 4B). No changes in *Il1b* were observed due to MI in either sex. However, *Il6* was robustly increased at all time points in females, while it was only increased at D56 in males. At D1, *Il6* was significantly increased in both sexes as well, but to a higher degree in males (Supplementary Figure S5). *Ccl2* was also increased in females at D7 and D28, returning to basal levels by D56, while its expression changed little in males. *Tnf* was not significantly affected by MI, but it was lower in males at D7 and D56 compared with females. *Ifng* was decreased in females at D28 but not males, and was decreased in both sexes at D56. *Il18* decreased at D7 in both sexes, returned to basal levels in males but remained decreased in females at D28, and increased only in females at D56.

### Changes in splenic cytokines after MI

**Figure 5:**
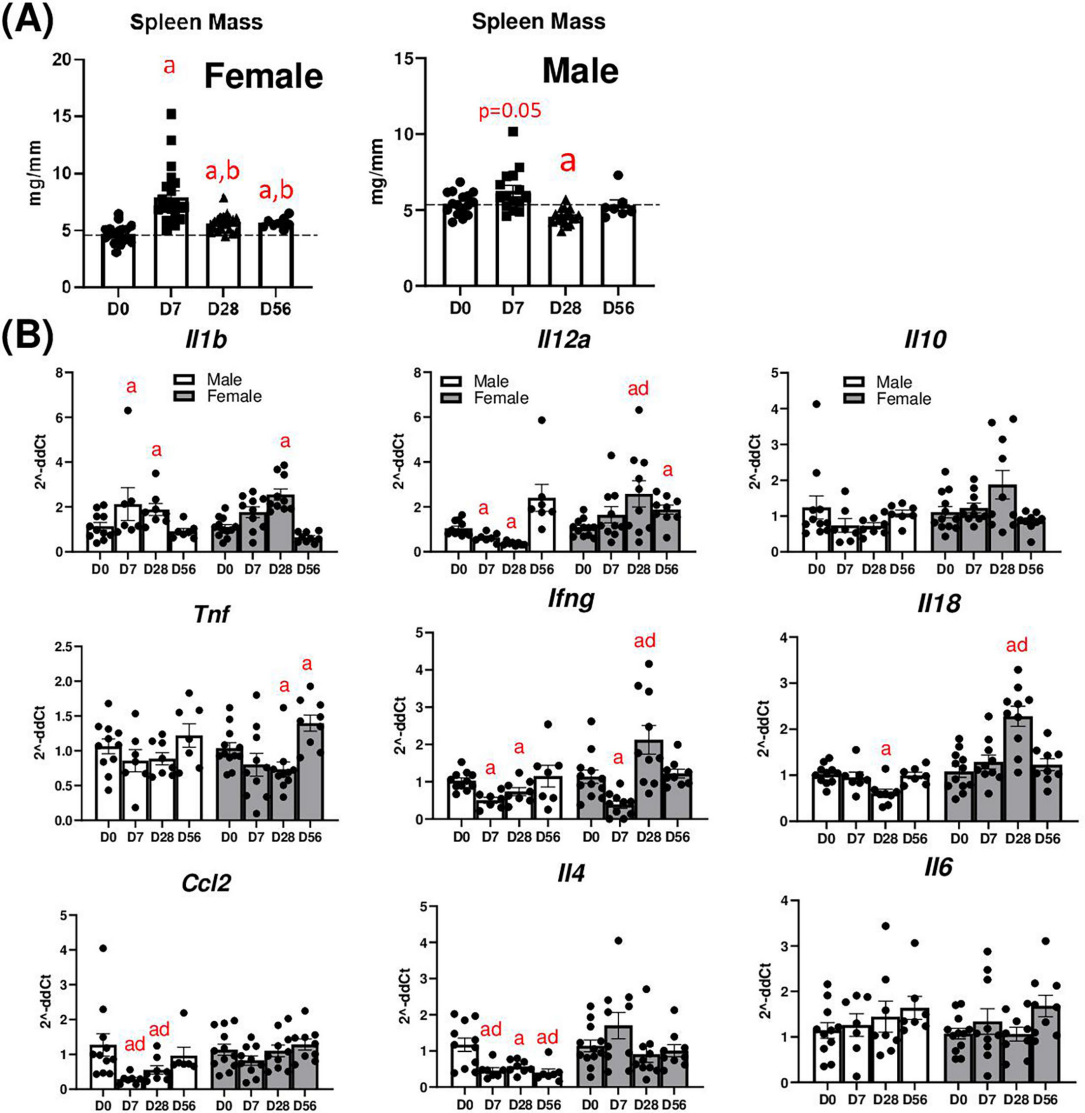
(**A**) Spleen mass after MI. (**B**) Gene expression analysis of inflammatory cytokines in the spleen after MI. a*: P*<0.05 vs. day D0, d: *P*<0.05 male vs. female. *N* = 7–15 mice per group.

The spleen is known to play a critical role in immune function and trafficking after MI and also plays important roles in autoimmunity [25,26,29]. We found that spleen mass was elevated at all time points after MI in females, peaking at D7 (Figure 5A). However, spleen mass did not change at D7 in males and was decreased at D28 and 56. We also assessed changes in cytokines in the spleen at these time points (Figure 5B). In females, *Il1b* increased at D28, but decreased by D56, while in males, no major changes in *Il1b* were observed. *Il12a* increased in females by D28 but decreased in males at D7 and 28; in both sexes, *Il12a* was increased at D56. *Il10* was not significantly altered after MI but was significantly higher in females than males at D28. *Tnf* decreased in females at D28 but was increased at D56; no changes were observed in males. *Ifng* decreased at D7 in both sexes, but was increased at D28 in females, and returned to basal levels at D56 in both sexes. *Il18* was increased at D28 in females, but decreased at D28 in males, then returned to basal levels by D56 in both sexes. *Ccl2* was decreased in males at D7 but unchanged in females. *Il4* decreased in males at all time points, but was unchanged in females. No changes in *Il6* in either males or females were observed. At D1 post-MI, splenic *Il4* and *Ifng* were also decreased in males, but not females (Supplementary Figure S6).

### MI leads to LV IgG and IgM deposition

**Figure 6:**
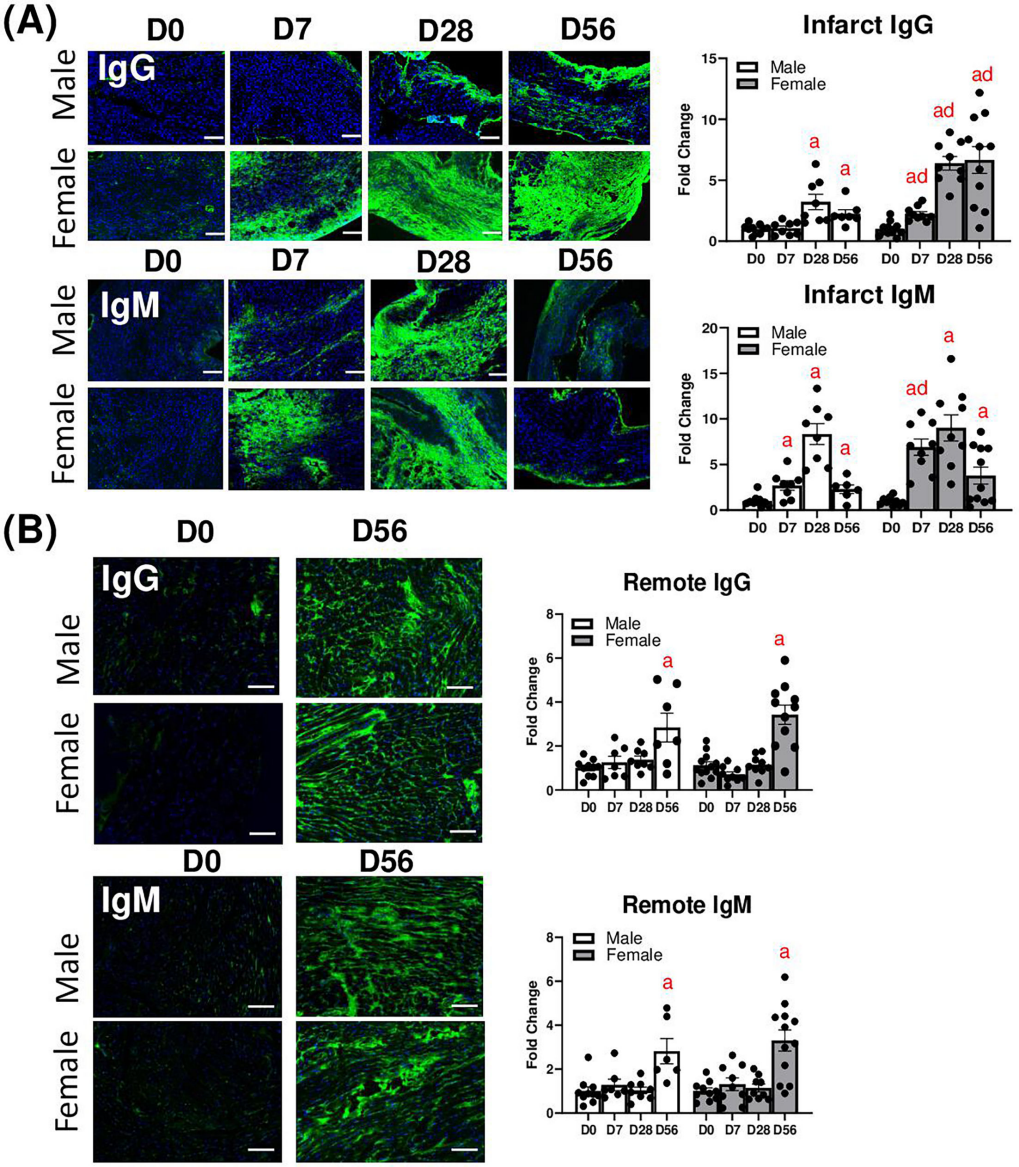
(**A**) IgG deposition (green) in the LV infarct and remote area. (**B**) IgM deposition in the LV infarct and remote area. a: *P*<0.05 vs. day D0, d: *P*<0.05 male vs. female. *N* = 7–15 mice per group.

We then tested whether IgG and IgM deposition was increased in the heart after MI by immunofluorescence microscopy (Figure 6). We found that IgG was increased in the infarct region in females as early as D7, continued to increase at D28, and remained elevated at D56. In males, IgG was elevated at D28 and D56, but overall, the staining was lower in males compared with females. IgM was increased in the infarct area in both males and females at D7, but more so in females, and was increased to similar levels in both sexes at D28. IgM decreased from D28 to D56 but remained elevated relative to D0. In the remote area, IgG was decreased at D7 in females and was increased in both sexes at D56 to a similar degree. IgM in the remote area was not changed in either sex at D7 or 28 but was increased by D56 post MI compared with D0.

### MI alters circulating levels of cytokines and autoantibodies

**Figure 7:**
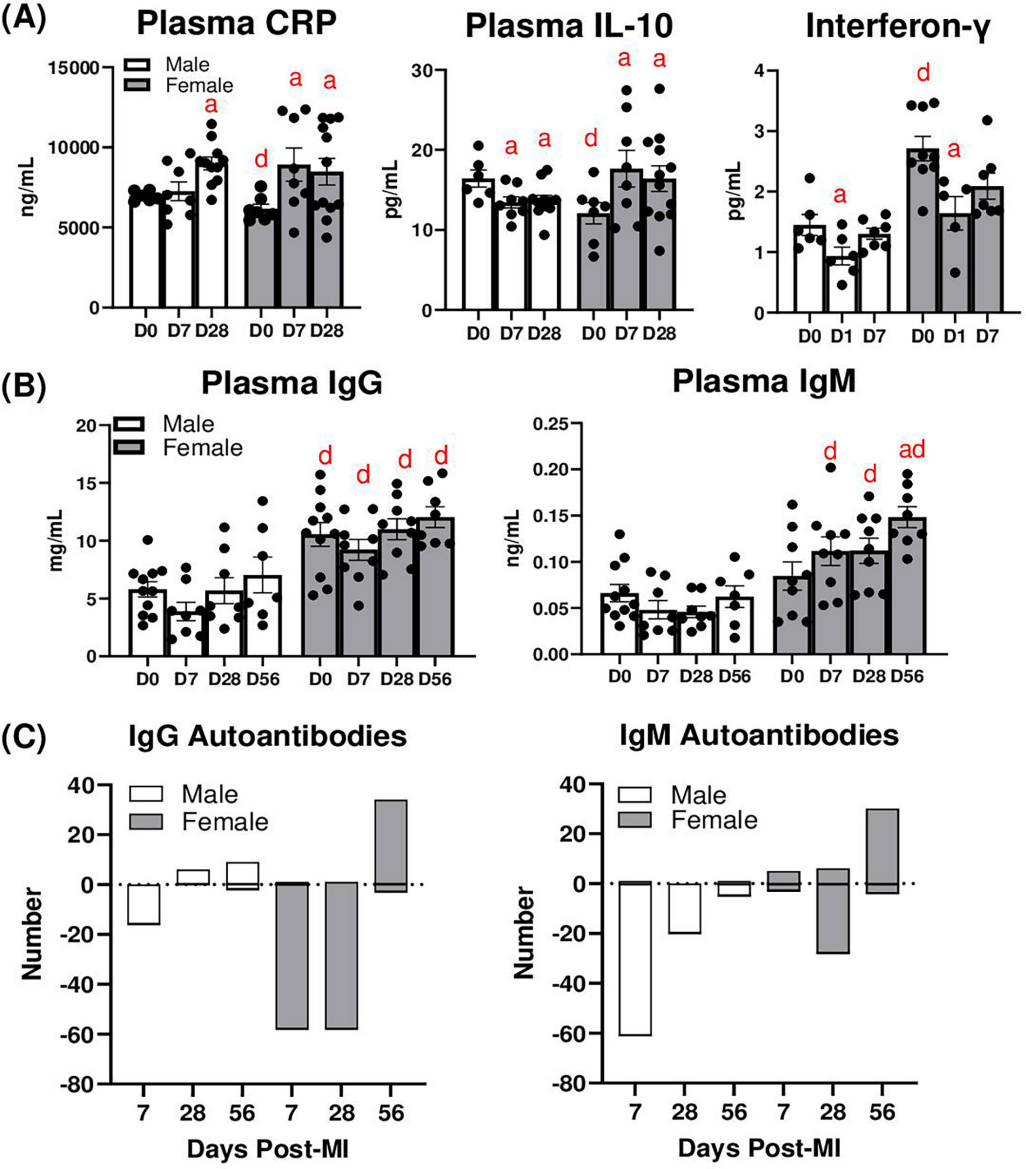
Changes in plasma cytokines and antibodies. (**A**) Plasma levels of CRP, IL-10, and IFN-γ. Plasma levels of (**B**) total IgG and IgM. (**C**) Numbers of autoantibodies either increased or decreased after MI. a: *P*<0.05 vs. day D0, d: *P*<0.05 male vs. female. *N* = 7–15 mice per group.

We measured circulating levels of cytokines, including CRP, IL-10, and IFN-γ in male and female mice post-MI. Plasma CRP was elevated in females at D7, and in males as well as in females at D28 (Figure 7A). Plasma IL-10 was decreased in males at D7 and D28 but increased in females at these time points (Figure 7A). Plasma IFN-γ was decreased at D1 in both sexes (Figure 7A), which may be due to decreased circulating lymphocytes at D1 (Supplementary Figure S7); however, there were no differences detected at D7. We then quantified levels of circulating IgG and IgM (Figure 7B), as well as IgG and IgM autoantibodies using an autoantigen array (Tables 1 and 2). Plasma IgG was higher in females at baseline and remained higher than males at all time points after MI; however, plasma IgG concentrations were unaffected by MI in either males or females. Total plasma levels of IgM were not different between males and females at baseline (Figure 7B), but they were significantly higher in females compared with males D7 and D28. Circulating IgM was increased at D56 in females relative to D0. Circulating autoantibodies were then measured by autoantigen array (Figure 7C). At D7, IgG autoantibodies against gDNA were elevated in females (compared with D0 controls), while no autoantibodies were increased in males (Table 1). Autoantibodies against 16 autoantigens were decreased in males, and autoantibodies against 58 autoantigens were reduced in females (Table 3). At D28, IgG autoantibodies against four different antigens (U-snRNP B/B′, LC1, H/K + ATPase, and HSPG) were elevated in males, versus only 1 in females (lysozyme), while 58 autoantibodies were decreased in females (Table 3). The biggest difference in autoantibodies was observed at D56, in which IgG autoantibodies against 34 different targets were elevated in females, relative to 9 in males, while 2 were decreased in males and 3 decreased in females. At D7 (Table 2), IgM autoantibodies against 5 targets (histone H2a, histone H1, ssDNA, gDNA, and lysozyme) were increased in females, versus 1 in males (lysozyme); 61 autoantibodies were decreased in males versus only 3 in females (Table 4). At D28, IgM autoantibodies against 6 targets were elevated in females (histone H2a, histone H1, ssDNA, gDNA, lysozyme and Factor H), while none were increased in males; 20 were decreased in males versus 28 in females. At D56, IgM autoantibodies against 30 targets were detected in females, versus 1 in males (DFS70); 5 were decreased in males versus 4 in females. These data indicate that MI alters levels of circulating IgG and IgM autoantibodies, with females having higher levels of autoantibodies at D56.

**Table 1:** Plasma IgG autoantibodies significantly elevated after MI (compared with D0 controls)

Male D7	Female D7	Male D28	Female D28	Male D56	Female D56
None	gDNA	U-snRNP B/B′ LC1 H/K + ATPase HSPG	lysozyme	Collagen I Collagen II Collagen IV Collagen V dsDNA gDNA M2 PR3 ssDNA	ACE2 BCOADC-E2 CENP-A Collagen III EJ GAD65 Gp210 HSPG IA-2 IF Jo-1 La/SS-b LC1 LKM1 Lysozyme MDA5 Mi-2 Mitochondrion MPO Myosin Nrp1 Nup 62 NXP2 P0 PCNA PL-12 PM/Scl-75 PR3 SAE1/SAE2 SLA/LP SP100 SRP54 Thyroglobulin TG

*N* = 6–9 per group.

**Table 2:** Plasma IgM autoantibodies significantly elevated after MI (compared with D0 controls)

Male D7	Female D7	Male D28	Female D28	Male D56	Female D56
lysozyme	Histone h2a Histone h1 ssDNA gDNA lysozyme	none	Histone h2a Histone h1 ssDNA gDNA Factor H lysozyme	DFS70	ACE2 BAFF Complement C9 EJ Factor P GAD65 Gp210 HSPG IA-2 IL-6 JO-1 La/SS-B LKM1 MBP Mi-2 Mitochondrion Myosin Nucleosome Nup62 P0 P1 PDC-E2 PL-7 PM/Scl-75 PR3 SP100 Thyroglobulin TIF1 gamma TPO tTG

*N* = 6–9 per group.

**Table 3:** Plasma IgG autoantibodies significantly decreased after MI (compared with D0 controls)

Male D7	Female D7	Male D28	Female D28	Male D56	Female D56
Alpha-fodrin BMP Calprotectin/S100 Collagen III DFS70 Gliadin Histone Nucleolin Nucleosome P2 SmD1 Smd2 Smd3 SRP54 Tau U1-snRNP C	Albumin alpha-fodrin Amyloid-beta BCOADC-E2 BPI CENP-A CENP-B Complement C4 Complement C5 Complement C9 CRP DFS70 EJ Factor B GAD65 H+/K + ATPase HSPG IF IFN-gamma IL-12 Jo-1 KU (P70/P80) La/SS-B La/SS-B Laminin LKM1 M2 MDA5 Mi-2 Myosin Nrp1 NXP2 OGDC-E2 P0 P1 P2 P2 PCNA PDC-E2 PM/Scl100 PM/Scl-75 PR3 Proteoglycan Prothrombin Ro/SS-A Ro/SS-A SAE1/SAE2 SP100 Thyroglobulin TLR4 TNF-alpha TPO tTG U1-snRNP A U1-snRNP C Vimentin Vitronectin	None	Albumin Amyloid beta [1–42] BPI CD4 CENP-A CENP-B Collagen I Complement C4 Complement C5 Complement C9 CRP DFS70 EJ GAD65 GBM gp210 HSPG IF Jo-1 KU (P70/P80) La/SS-B Laminin Lysozyme M2 MDA5 Mi-2 Mitochondrion Nrp1 Nucleolin Nup 62 OGDC-E2 P0 P1 P2 PM/Scl 100 PM/Scl-75 PR3 Proteoglycan Prothrombin Ro/SS-A(52 kDa) Ro/SS-A SAE1/SAE2 Scl-70 SLA/LP Sm/RNP SmD1 SP100 SRP54 Thyroglobulin TIF1 gamma TLR4 TNF-alpha TPO tTG U1-snRNP U1-snRNP C U-snRNP B/B′ Vimentin	Nucleosome MDA5	Histone Histone H2A Factor P

*N* = 6–9 per group.

**Table 4:** Plasma IgM autoantibodies significantly decreased after MI (compared with D0 controls)

Male D7	Female D7	Male D28	Female D28	Male D56	Female D56
ACE2 Aggrecan Alpha fodrin Amyloid beta [1–42] AQP4 BAFF BCOADC-E2 BPI Calprotectin/S100 CD40 Collagen III Collagen IV Complement C5 Complement C6 CRP DFS70 Factor B Factor H Factor P Fibrinogen type I-S Fibronectin GBM Gliadin gp210 Histone Histone H2A HSPG IL-17A KU (P70/P80) LPS MBP Mi-2 Nucleolin Nup 62 OGDC-E2 P1 P2 PCNA PM/Scl 100 PM/Scl-75 Proteoglycan Prothrombin Ro/SS-A(52 kDa) Scl-70 Sm Sm/RNP SmD SmD1 SmD2 SmD3 SP100 SRP54 Tau Thyroglobulin TLR4 TPO U1-snRNP 68/70 kDa U1-snRNP A U1-snRNP C U-snRNP B/B′ Vimentin	CENP-A GAD65 PR3	Collagen IV Complement C1q Complement C6 Cytochrome C Factor B Factor H Factor I Factor P GBM Histone H2A HSPG IL-17A MPO Sm Sm/RNP SmD SmD1 SmD2 SmD3 U1-snRNP	Amyloid beta [1–42] CENP-A CENP-B DFS70 EJ Factor H GAD65 H/K-ATPase Histone H1 Histone H3 HSPG IFN-gamma IL-6 MIgControl 1 Nrp1 OGDC-E2 PR3 Proteoglycan Ro/SS-A(52 kDa) SP100 SRP54 ssDNA Thyroglobulin TLR4 TPO U1-snRNP 68/70 kDa U1-snRNP A U-snRNP B/B'	AQP4 Collagen V Histone Histone H2A Nucleosome	BPI Factor P Histone H2A MBP

*N* = 6–9 per group.

### Discussion

In the present study, we assessed both the acute and chronic inflammatory response to MI in the heart, spleen, and peripheral blood. Our study demonstrates that there are robust sex differences in post-MI acute and chronic remodeling, the local and systemic inflammatory response, and development of autoreactive antibodies (Table 2). Our study suggests that MI may induce a chronic autoimmune response, particularly in females. These results have critical implications for the clinical scenario, as female MI patients are more likely to develop autoimmune disorders which may affect cardiovascular disease outcomes [30].

To our knowledge, this is the first study to comprehensively assess sex differences in the chronic local and systemic inflammatory and autoimmune response to MI. Previous studies have demonstrated that males have decreased survival during the first week after MI and worse LV function in the chronic phase [31], which is consistent with our findings. Males also have higher levels of neutrophil infiltration in the infarct area [31,32], which was true in our study as well. Another study demonstrated that females had higher levels of myocardial TNF-α and IL-1β after MI, despite better functional and survival outcomes [33]. Although we did not see changes in these cytokines, we found that IL-6 was markedly elevated at D7 in females and remained elevated in the remote area.

We also found that females had higher levels of leukocytes in the heart after MI, which appears to be due to higher levels of both T and B cells; this corroborates findings in human patients demonstrating that females with ST-elevation MI (STEMI) have higher levels of circulating leukocytes, as well as lymphocyte/monocyte ratios than men [34]. We observed elevated levels of plasma CRP in females as early as D7, and in males and females at D28, indicative of systemic inflammation [35]. In addition to higher levels of pro-inflammatory cytokines such as *Il6* and *Ccl2* being expressed in the hearts of females, we found that the anti-inflammatory cytokine *IL-10* was robustly increased in the infarct area of females compared with males at D7. IL-10 administration improves post-MI outcomes in mouse models [28,36]; however, mice lacking the Il10 gene do not exhibit impaired cardiac remodeling or worse post-MI outcomes [37]. IL-10 is produced acutely following MI, and its fluctuations follow a similar pattern to other pro-inflammatory cytokines [28], which we found to be true in our study as well. Thus, the increase in *Il10* that we observed in the female heart could be a compensatory response to increased levels of pro-inflammatory cytokines, including *Il6* and *Ifng*. Another component of the increased *Il10* response in females could be the increased numbers of immune cells in the post-MI female heart at D7, particularly T cells, which are a major source of IL-10 [38]. We also observed sex differences in circulating levels of IL-10, with males having decreased levels and females having increased levels at D7 and D28. Again, this could reflect overall increased systemic inflammation, as plasma CRP was elevated at D7 in females.

T cells are known to play a complex role in modulating the inflammatory and remodeling response to MI. We found that CD3^+^ T cells expanded in the hearts of female mice at D7 after MI, while decreasing in males. During the clearance of necrotic cells post-MI, cardiac antigens are phagocytosed and then presented to T cells in the heart-draining mediastinal lymph nodes [39]. These cells then infiltrate the heart and participate in cardiac inflammation and repair. In nonobese diabetic (NOD) mice, which are prone to autoimmune development after MI, inflammatory monocytes present cardiac myosin to T cells, promoting the development of myosin-specific T cells [40,41]. Specific T helper (Th) subsets have been studied in the context of MI, with each subset secreting cytokines that play distinct roles in inflammation, remodeling, and repair. Th1 cells are a major source of IFN-γ [42], which we found to be higher in females at D7. Higher levels of T cells and lower levels of neutrophils could be one mechanism by which females display improved cardiac remodeling compared with males, but it could also render females more prone to autoimmune development.

B cells also play a critical role in regulating the inflammatory and remodeling response to MI. Some B cell subsets can be protective in MI remodeling by inducing neutrophil apoptosis [43], which may partially explain why we observed increased levels of cardiac neutrophils in males, as cardiac B cells were lower in male mice. On the other hand, studies in C5BL/6 J mice showed that cardiac B cells express CCL7 and recruit Ly6C^hi^ monocytes to the heart, which contribute to tissue injury and impaired heart function [5]. Studies in NOD mice indicate infiltration of B and T cells in the heart after MI, which is associated with the development of autoantibodies against cardiac myosin heavy chain [44]. We found that B cells were decreased in male hearts at D7, while they remained unchanged in females. By D56, cardiac B cells were increased in the infarct area in both males and females, but to a higher extent in females; in the remote area, B cells were increased at D56 in females only. These data indicate that there are temporal changes in B cell dynamics that likely play a role in cardiac inflammation, remodeling, and repair. In addition, immunofluorescence microscopy indicated IgG and IgM deposition in the remote area of the heart at D56 in males and females. These data corroborate findings in human HFrEF patients in which increased levels of IgG antibodies are found in the heart [45]. We also found higher levels of circulating lymphocytes and B cells in females at D56, which was associated with elevated levels of plasma IgM, IgG, and IgG and IgM autoantibodies. Thus, females may be more prone to an aberrant humoral response after chronic MI, which contributes to autoimmune development.

The spleen plays a critical role in initiating the inflammatory response to MI. We observed changes in spleen mass after MI, in which female spleens were larger at all time points analyzed, while male spleens tended to be smaller at D28 and D56 compared with D0 mice. Previous reports indicate that the spleen is enlarged at D5 and D7 post-MI in males, in association with expansion of neutrophils and monocytes [46,47]. While we saw a trend for male spleen weight to be increased at D7 (*P*=0.05), the effect was much larger in females, and male as well as female spleens exhibited increased neutrophil and monocyte expansion at D7. Despite splenic neutrophils and monocytes returning to basal levels by D28, female spleens remained enlarged at D28 and D56, which could be due to higher numbers of splenic B and T lymphocytes in females. Males had lower levels of splenic B cells than females at D28. Recent studies indicate that the spleen is a major site of antigen processing and MHC class II mediated presentation in B cells after MI [48]. Thus, splenic B cells in females may be more active in presenting antigens to CD4^+^ and CD8^+^ T cells after chronic MI. In support of this concept, females also had higher levels of splenic T cells at D28 and 56, including both CD4^+^ and CD8^+^ T cells. Splenic CD4^+^ T cells (i.e. helper T cells) have been demonstrated to promote adverse remodeling after MI [49,50] and play a role in activating autoimmune responses to cardiac antigens [41]. CD8^+^ T cells also promote cardiac injury after MI [9,10], indicating that females may be more susceptible to T cell-mediated cardiac injury.

Splenic cytokines were also differentially expressed in males and females after MI. IL-1β transcript increased in female spleens at D7 and in male and female spleens at D28. IL-1β most importantly plays a role in innate immunity by activating a pro-inflammatory state in monocytes and neutrophils [51] and also promotes activation of antigen processing in lymphocytes such as B cells and CD4^+^ T cells [51,52]. TNF-α, which was increased at D56 in females, supports the formation of lymphoid follicles and formation of memory B and T cells to antigens [53,54]. At D28 in female mice, there were increased levels of IL-12a, IFN-γ, and IL-18. IL-18 plays a pro-inflammatory role by supporting T cell IFN-γ and TNF-α production and suppressing IL-4 production [55,56]. Specifically, IL-18, in the presence of IL-12, acts on Th1 cells, macrophages, dendritic cells, among other cell types, to promote IFN-γ production. The increase in these cytokines at D28 suggests increased Th1 cells in female mice. Mice that lack B and T cells exhibit decreased inflammation following MI, and reconstitution with CD4+T cells reverses this phenotype [57]. Overall, the splenic cytokine profile in females reflects an elevated pro-inflammatory/Th1 dominated immune response.

The most striking sex difference that we observed in post-MI autoimmune signatures was the rise in circulating total immunoglobulins and autoantibodies, particularly in females. Females had higher total IgG levels at all time points and higher IgM at D7, D28, and D56. In C57BL/6 mice, immunoglobulin concentrations increase with age, with females typically having higher IgG and IgM concentrations [58,59]. In early studies in MI patients, Ebringer et al. showed a fall in serum IgG levels in the first five to seven days after MI, and then a subsequent elevation above control levels, suggesting that the tissue damage leads to the presence of antigens that can lead to an autoimmune-like response [60].

Increased levels of circulating autoantibodies are hallmarks of autoimmune disease in both humans and animal models [61,62]. In D7 females, we observed increases in IgG autoantibodies against genomic (g) DNA, and IgM autoantibodies against gDNA, single stranded (ss) DNA, and histones H1 and H2A. Antinuclear IgG antibodies, which are autoantibodies that target nuclear components such as DNA, RNA, and nuclear protein complexes (i.e. histones), are the most common class of autoantibodies that are used to define and diagnose autoimmune conditions such as systemic lupus erythematosus (SLE) [63]. Extracellular DNA and histones act as damage-associated molecular patterns during myocardial ischemic injury, thus initiating toll-like receptor (TLR)-mediated signaling cascades in antigen-presenting cells such as macrophages, which could then initiate production of autoimmune responses against these molecules [64]. Autoantibodies against these nuclear components remained elevated at day 28 in females, indicating a persistent antinuclear autoantibody response. At day 56, several other antinuclear antibodies were observed in females, including anti-CENP-A (IgM), Gp210 (IgG and IgM), Mi-2 (IgG and IgM), NXP2 (IgG), PCNA (IgG), PM-Scl75 (IgG and IgM), Sp100 (IgG and IgM), nucleosome (IgM), and TIF1 gamma (IgM). Antinuclear antibodies were also increased in males, albeit to a lesser degree. In day 56 males, IgG autoantibodies against nuclear components such as gDNA, ssDNA, and dsDNA were increased. In day 7 males, IgG autoantibodies against U-snRNP B/B′ were increased. Autoantibodies against U-snRNPs, including B/B′, are a class of RNA-associated autoantigens and are a feature of SLE and mixed connective tissue diseases [65]. Anti-DFS70 IgM, another nuclear antigen observed in several inflammatory and autoimmune conditions [66], was the only IgM autoantibody elevated in day 56 males. The only IgM autoantibody elevated in day 7 males was against lysozyme, against which IgG autoantibodies also developed in day 28 females. Lysozyme is a major component of specific neutrophil granules [67], and autoantibodies against lysozyme are strongly associated with inflammatory rheumatic conditions [68]. Anti-IgM responses against lysozyme against males, but not females, at day 7 could reflect the higher myocardial neutrophil numbers that we observed in males at this time point.

By day 56, we observed increased autoantibodies against several antigens involved in cardiac function and remodeling, including several collagen isoforms. In males, IgG autoantibodies against collagens I, II, IV, and V were detected. Collagen I is the major cardiac interstitial collagen and forms the majority of scar tissue after MI [69], and autoantibodies against collagen I have been detected in SLE patients [70]. Increased autoantibodies against collagen IV, the major constituent of the basement membrane, have been detected in MI patients regardless of other risk factors [71]. Females only had autoantibodies to collagen III (IgG), which may be due to the fact that female hearts express higher levels of this isoform than males [72].

We also observed increased IgG and IgM autoantibodies against proteins/components of the cardiac myocytes in females at day 56, including ACE2, mitochondria, and myosin. While ACE2 is predominantly expressed in the lung, it is also expressed in the heart, where it plays a protective role against various cardiac insults, including MI [73]. Anti-mitochondrial antibodies have been well characterized in autoimmune disorders, including SLE [74], and have also been detected in MI patients [75]. Anti-myosin antibodies have been well documented in MI and heart failure patients [14,76], and immunization with purified myosin is a standard method of inducing myocarditis in mouse models [77]. It will be important to assess the dynamics of the IgG and IgM autoantibody responses in future studies. The majority of known pathogenic autoantibody responses are mediated by IgG. IgM autoantibodies are primarily produced by B1 B cells (~80% in the mouse) that reside in the pleural and peritoneal cavities and have a low affinity and broad specificity for self-antigens. These antibodies have been shown to mediate protective roles, such as clearance of apoptotic cells in healthy states and dampening inflammatory responses in inflammatory and autoimmune diseases [78].

Although circulating IgG and IgM autoantibodies and total IgM were increased at D56, we found that MI *decreased* circulating levels of several autoantibodies at D7 and D28. For both males and females, there was a robust shift towards fewer circulating IgG autoantibodies at the D7 time point, which was also true at D28 for females. One potential reason for this is the impairment in lymphocyte production after MI [27], as we observed reductions in circulating, splenic, and cardiac B cells at D7 and D28. However, the circulating and cardiac B cell population expanded in females at D56, coinciding with a shift towards increased levels of circulating IgG and IgM autoantibodies. Thus, acute MI remodeling (up to D28) may be associated with impaired antibody responses, while chronic MI (D56) is associated with increased B cell and autoantibody production.

The increased numbers of IgG and IgM autoantibodies in the plasma in females were associated with higher levels of antibody deposition in the infarct area, particularly IgG, which plateaued by D28 and D56. However, levels of IgG and IgM in the LV remote were similar between sexes and did not increase until D56. Thus, the link between circulating autoantibodies and cardiac IgG/IgM deposition remains to be determined.

One important question that our study raises is the link between the acute inflammatory response and the innate immune response following MI. Although our array did not detect anti-troponin antibodies, previous studies have reported positivity for anti-troponin antibodies after MI in BALB/c mice [79]. Since we observed higher levels of plasma troponin in females 24 hr after MI, acute release of cardiac proteins and other potential autoantigens represent one potential mechanism by which females are more susceptible to post-MI autoimmune responses. Another potential mechanism is antigen presentation by antigen-presenting cells such as monocytes or dendritic cells, which present autoantigens such as myosin to T cells in the mediastinal lymph node following MI to promote their activation [80]. Previous studies found that females have higher levels of cardiac dendritic cells at D5 post-MI, which remain elevated by D56 relative to males [33]. Thus, heightened activation of dendritic cells which present antigen to adaptive immune cells is another potential mechanism linking the acute inflammatory response to autoimmune activation in females.

### Limitations

There are some limitations of our study. We only focused on autoantibodies of the IgG and IgM isotypes, which are responsible for the majority of autoimmune responses; however, certain conditions can involve IgA responses [81]. Furthermore, it will be important to assess IgG isotype-specific (IgG1, IgG2(abc), IgG3, IgG4) responses in future studies. We also focused primarily on cardiac and secondary lymphoid organ (splenic) responses; however, it is possible that other organs/tissues are affected by MI-induced autoimmune responses. In particular, nuclear and mitochondrial antigens are ubiquitously expressed throughout the body, and it remains to be determined whether other tissues are affected by autoantibodies against these targets after MI. Furthermore, while we detected increased IgG and IgM in the heart, we did not determine which specific antigens these antibodies were binding, although the circulating autoantibodies gave us some potential targets.

## Conclusions

In conclusion, our data indicate that despite similar infarct sizes and degree of remodeling, the inflammatory response is exacerbated in females after MI and involves an increased contribution from the lymphocytic compartment. Furthermore, both males and females develop an autoimmune response, which is greater in females. Our study suggests that females are more susceptible to autoimmune-like signatures after MI and may benefit from sex-specific therapy targeting the inflammatory/autoimmune response.

**Clinical Perspectives**

- Autoimmune disease is highly prevalent in women and linked with adverse MI outcomes, but sex differences in the chronic autoimmune response following myocardial infarction (MI) in mouse models have not been characterized.
- Despite similar functional outcomes and better survival, females exhibit elevated inflammatory responses and persistence of local and systemic autoimmune signatures after MI.
- Females may be at higher risk of developing autoimmune-mediated cardiac injury and systemic autoimmunity after MI, and therapies targeting these pathways may be more efficacious in females.

## Supplementary material

Online supplementary material

## Data Availability

All materials, data, and protocols are present in the manuscript or are available upon request.

## Abbreviations

LV: left ventricle
MI: myocardial infarction
PBL: peripheral blood leukocyte
SLE: systemic lupus erythematosus
