# Supplementary material for: Sex differences in the chronic autoimmune response to myocardial infarction
Source: Clin Sci (Lond). 2025 Jun 17;139(12):627–48. doi: 10.1042/CS20243091 (PMC12238818; doi:10.1042/CS20243091)
Supplement: Online supplementary material [file cs-139-12-CS20243091-supp1.docx]

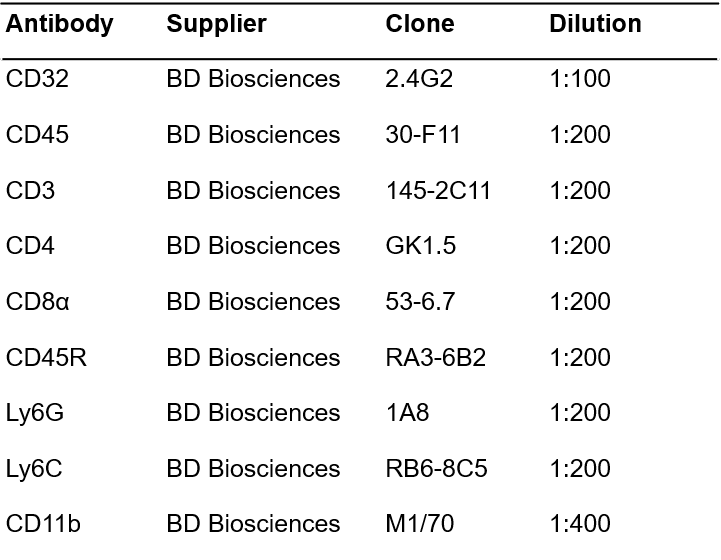


**Table S1. Monoclonal antibodies used for flow cytometry.**

**Supplemental Figure 1.** Morphometric parameters following MI—LV mass and lung mass normalized to tibia length in (A) males and (B) females. a—p<0.05 vs. day (D) 0, b—p<0.05 vs. D7, c—p<0.05 vs. D28, d—p<0.05 male vs. female. N=7-15 mice per group.

**(B)**

**(A)**

a

a

a

a,b

a

a

a,b,c

a

a

a

female

male


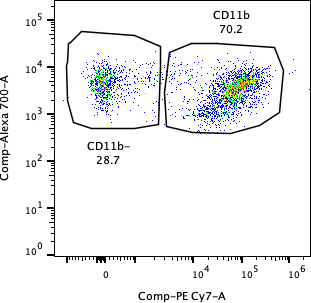

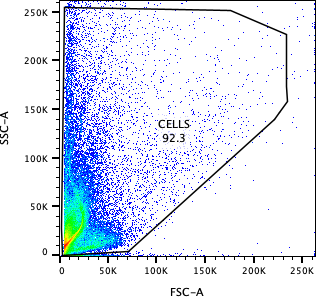

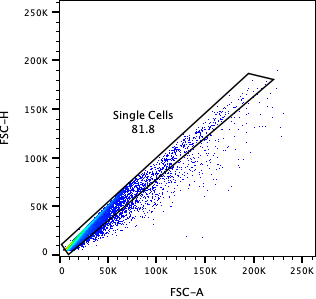

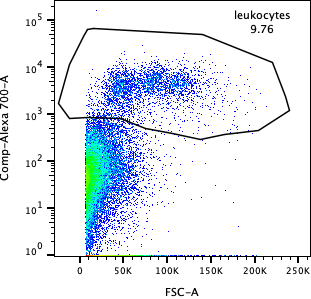


FSC-A

FSC-A

FSC-A

SSC-A

FSC-H

CD45-AF700


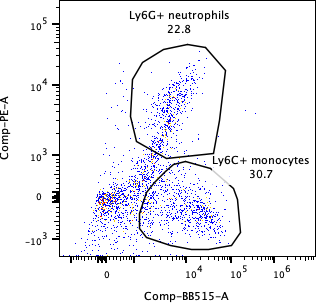


CD45-AF700

CD11b-PE-Cy7

Ly6G-PE

Ly6C-FITC


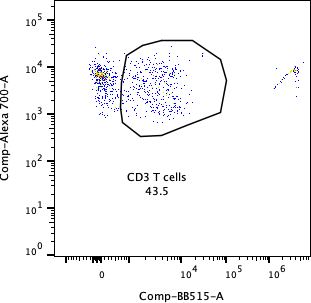


CD3-BV650

CD45R-APC

CD45-AF700


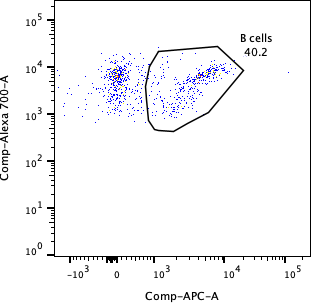


CD45-AF700

**Supplemental Figure 2.** Representative flow cytometry gating strategy for heart tissue (D7 post-MI). Data were acquired using a BD FACSymphony A3 and analyzed using FlowJo.


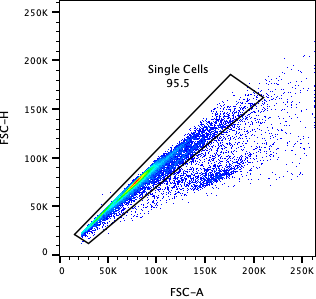

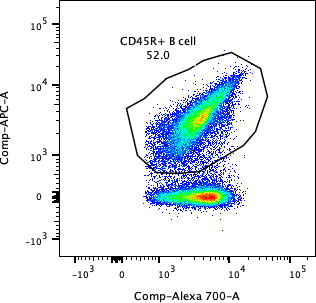

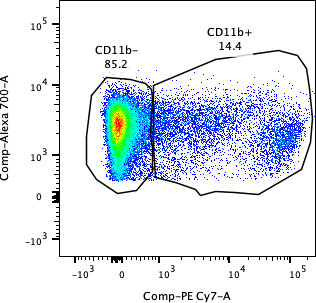

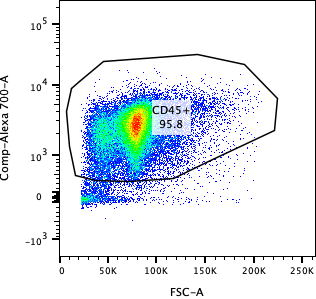

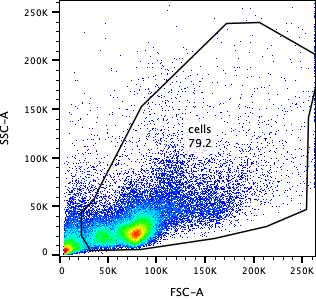


FSC-A

FSC-A

FSC-A

SSC-A

FSC-H

CD45-AF700

CD45-AF700

CD11b-PE-Cy7

Ly6G-PE

Ly6C-FITC

CD3-BV650

CD45R-APC

CD45-AF700

CD45-AF700

**Supplemental Figure 3.** Representative flow cytometry gating strategy for spleen. Data were acquired using a BD FACSymphony A3 and analyzed using FlowJo.


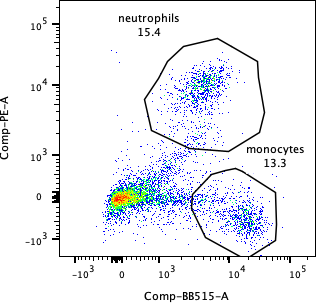

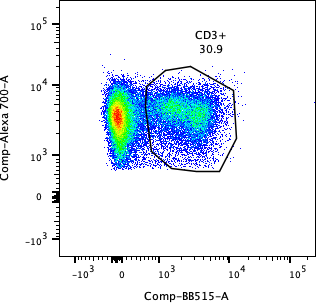

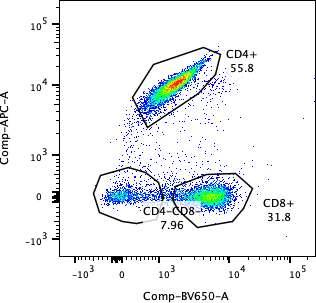


CD8-BB700

CD4-BV421


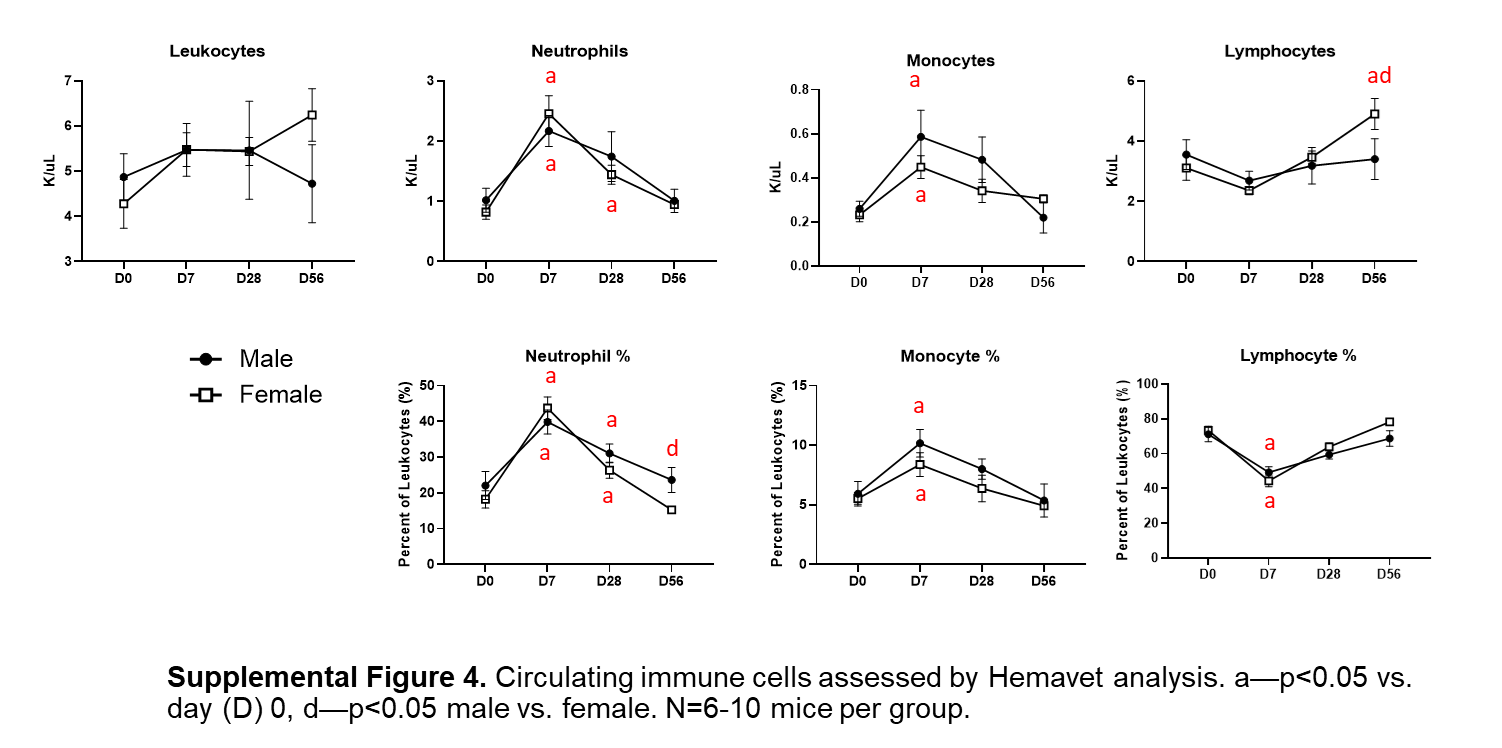


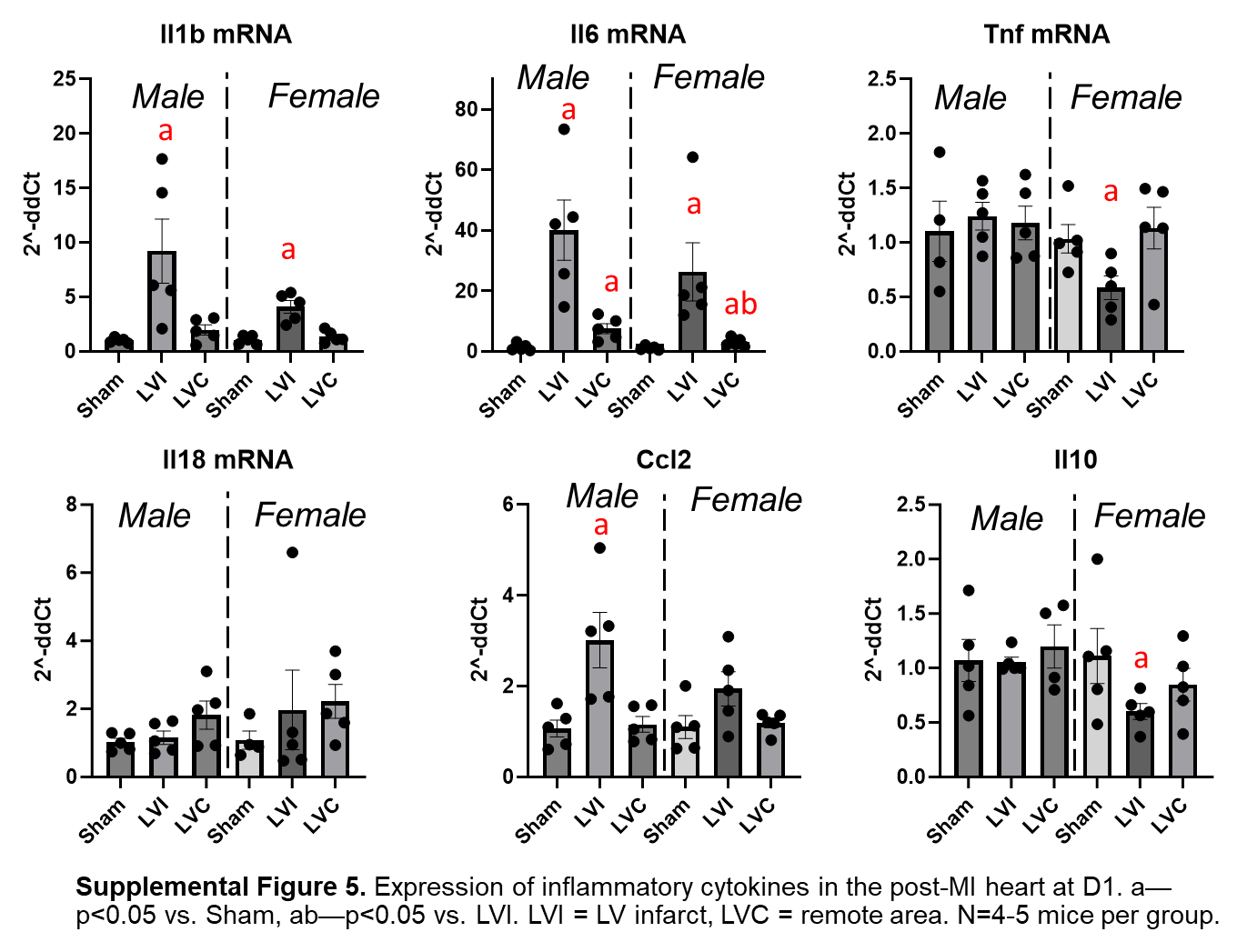


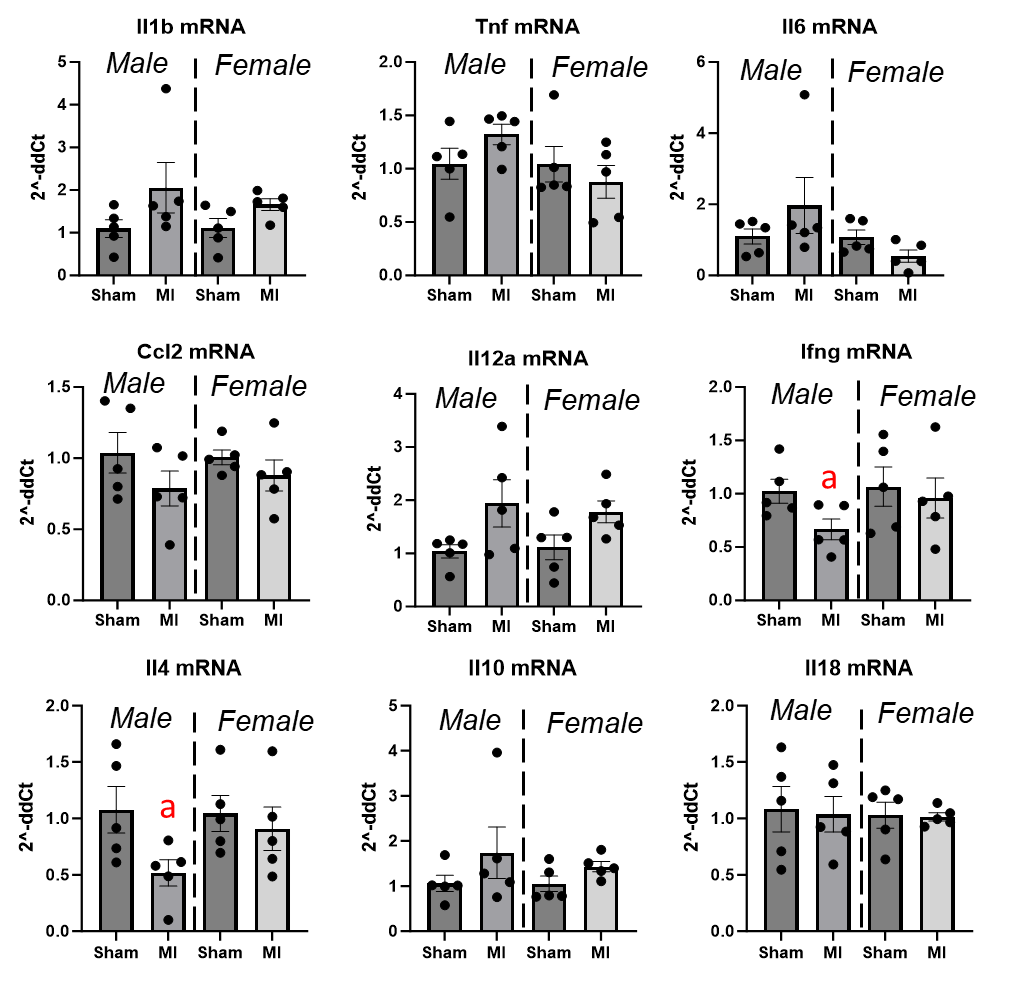


**Supplemental Figure 6.** Expression of inflammatory cytokines in the spleen at D1 post-MI. a—p<0.05 vs. Sham. N=4-5 mice per group.
